# Validation of ctDNA Quality Control Materials Through a Precompetitive Collaboration of the Foundation for the National Institutes of Health

**DOI:** 10.1200/PO.20.00528

**Published:** 2021-06-01

**Authors:** P. Mickey Williams, Thomas Forbes, Steven P. Lund, Kenneth D. Cole, Hua-Jun He, Chris Karlovich, Cloud P. Paweletz, Daniel Stetson, Laura M. Yee, Dana E. Connors, Susan M. Keating, Benoit Destenaves, Megan H. Cleveland, Christie J. Lau, J. Carl Barrett, Gary J. Kelloff, Robert T. McCormack

**Affiliations:** ^1^ Molecular Characterization Laboratory, Frederick National Laboratory for Cancer Research, Leidos Biomedical Research Inc, Frederick, MD; ^2^ National Institute of Standards and Technology, Gaithersburg, MD; ^3^ Medical Oncology, Dana-Farber Cancer Institute, Boston, MA; ^4^ Belfer Center for Applied Cancer Science, Dana-Farber Cancer Institute, Boston, MA; ^5^ Translational Medicine, Oncology R&D, AstraZeneca, Waltham, MA; ^6^ Division of Cancer Treatment and Diagnosis, National Cancer Institute, National Institutes of Health, Bethesda, MD; ^7^ Foundation for the National Institutes of Health, Bethesda, MD; ^8^ CCS Associates, San Jose, CA; ^9^ National Cancer Institute, Rockville, MD; ^10^ Independent

## Abstract

We report the results from a Foundation for the National Institutes of Health Biomarkers Consortium project to address the absence of well-validated quality control materials (QCMs) for circulating tumor DNA (ctDNA) testing. This absence is considered a cause of variance and inconsistencies in translating ctDNA results into clinical actions.

**METHODS:**

In this phase I study, QCMs with 14 clinically relevant mutations representing single nucleotide variants, insertions or deletions (indels), translocations, and copy number variants were sourced from three commercial manufacturers with variant allele frequencies (VAFs) of 5%, 2.5%, 1%, 0.1%, and 0%. Four laboratories tested samples in quadruplicate using two allele-specific droplet digital polymerase chain reaction and three (amplicon and hybrid capture) next-generation sequencing (NGS) panels.

**RESULTS:**

The two droplet digital polymerase chain reaction assays reported VAF values very close to the manufacturers’ claimed concentrations for all QCMs. NGS assays reported most single nucleotide variants and indels, but not translocations, close to the expected VAF values. Notably, two NGS assays reported lower VAF than expected for all translocations in all QCM mixtures, possibly related to technical challenges detecting these variants. The ability to call *ERBB2* copy number amplifications varied across assays. All three QCMs provided valuable insight into assay precision. Each assay across all variant types demonstrated dropouts at 0.1%, suggesting that the QCM can serve for testing of an assay’s limit of detection with confidence claims for specific variants.

**CONCLUSION:**

These results support the utility of the QCM in testing ctDNA assay analytical performance. However, unique designs and manufacturing methods for the QCM, and variations in a laboratory’s testing configuration, may require testing of multiple QCMs to find the best reagents for accurate result interpretation.

## INTRODUCTION

Tumor biomarkers derived from liquid biopsies have substantially altered the treatment of patients with cancer and are redefining development of future therapies. Potentially informative cancer biomarkers derived from liquid biopsies include circulating tumor cells, tumor cell–derived exosomes, circulating nucleic acids, and normal blood elements altered by exposure to the tumor.^1-3^

**CONTEXT**

**Key Objective**
The analysis of circulating tumor DNA (ctDNA) is an area of intense interest for cancer patient management. ctDNA assays may permit early cancer detection, therapeutic targeting, response monitoring, resistance biomarker surveillance, and minimal residual disease detection. At present, there are no universally accepted quality control materials (QCMs) for ctDNA assays. Such materials are needed for establishing assay analytical claims. This effort provides the results of three different QCMs tested across five different assay platforms.
**Knowledge Generated**
The data presented demonstrate that all three QCMs can serve as needed materials for analytical performance testing of ctDNA assays. The QCM generated expected results, although several unexpected results occurred, such as next-generation sequencing assays, under-reporting expected variant allele frequencies of translocations.
**Relevance**
The QCMs are a valuable tool for exploring analytical performance of ctDNA assays. QCM will be available as manufactured reagents, which permit sufficient material to replicate testing of multiple variants.

Circulating tumor DNA (ctDNA) is a biomarker of prime interest for mutation-driven precision therapy. Potential clinical use of ctDNA has been demonstrated across multiple cancers, from early detection through metastatic disease management.^1,3-5^ Such diagnostic and therapeutic promise from a single class of biomarkers is unprecedented.

The confluence of new technologies and changing regulatory oversight has impeded the translation of ctDNA results into routine practice and patient benefit. For example, comparison studies among laboratories pointed toward technical issues and inadequate quality control for accurate result interpretation.^6,7^ This position was mirrored in a recent report from ASCO and College of American Pathologists (CAP).^8^

A public-private research partnership under the Foundation for the National Institutes of Health (FNIH) Biomarkers Consortium addressed this problem and identified the absence of well-validated quality control materials (QCMs) that facilitate accurate interpretation of ctDNA testing results as a root cause of the variance seen in interpreting and translating ctDNA results into clinical action.^8,9^ The team worked with three commercial manufacturers (Horizon Discovery Ltd, [Waterbeach, Cambridge, UK], Microgenics Corporation/Thermo Fisher Scientific [Fremont, CA], LGC SeraCare Life Sciences, Inc. [Milford, MA]) to source QCM, soliciting input on the selection of variants clinically relevant for cancer patient management from a large group of stakeholders, including National Cancer Institute (NCI), US Food and Drug Administration, National Institute of Standards and Technology (NIST), ASCO, CAP, Association for Molecular Pathology, and academic institutions. Table 1 depicts the list of variants. The variants were selected to represent those found in multiple tumor histologies and include four different variant classes (single nucleotide variants [SNVs], insertions or deletions [indels], translocations, and a copy number variant [CNV]). A study was designed to assess the QCM in three main components: determine the suitability of the QCM as aids in ctDNA assay analytical performance testing (phase I), functional characterization (phase II), and a clinical pilot to test their use as assay controls and generate real-world evidence across multiple commercial and academic clinical laboratories (phase III).

**TABLE 1.**
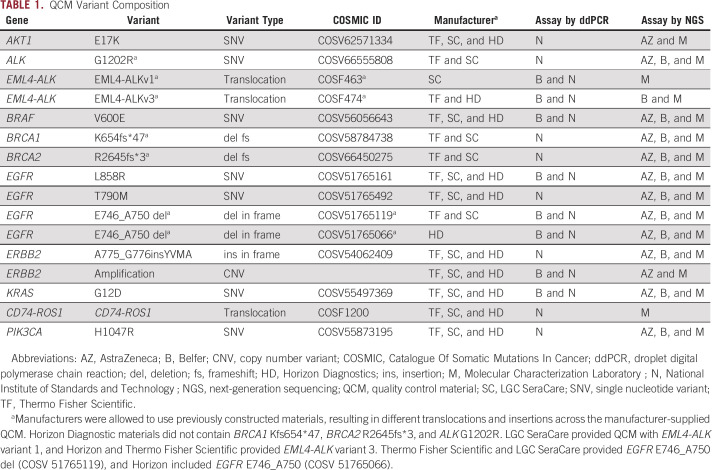
QCM Variant Composition

We here report the results of phase I of the three QCMs across four laboratories, using two droplet digital polymerase chain reaction (ddPCR) assays and three next-generation sequencing (NGS) panels (one amplicon-based and two hybrid capture). Each assay was developed by the laboratories to be fit for the purpose of detecting ctDNA. The five assays were performed as research assays and were not performed in CAP/Clinical Laboratory Improvement Amendments–accredited laboratories. Phase I experiments were the first step to support the overall goal of the project, which is to provide well-characterized QCM to assist in advancing the clinical validity and utility of ctDNA measurements.

## METHODS

**FIG 1.**
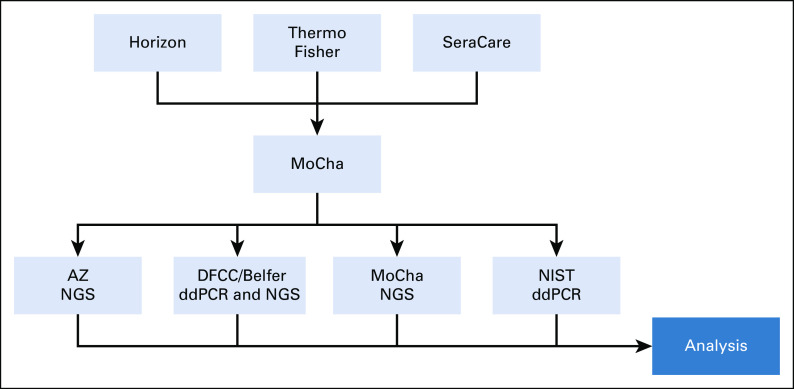
Performance evaluation study workflow. A central laboratory was used for preanalytics to minimize preanalytic variables. The QCMs from the three vendors—Horizon, LGC SeraCare, and Thermo Fisher Scientific—were sent to MoCha for extraction and then pooled for quality control testing. Samples were then diluted to 1 ng/mL, aliquoted into fresh tubes, and blinded for NGS, or partially blinded for ddPCR testing. The samples were then distributed to AZ, Belfer, MoCha, and NIST for assay using gene-targeted capture-based NGS (AZ), amplicon-based NGS (Belfer), targeted gene panel NGS (MoCha), and ddPCR platforms at Belfer and NIST. The use of the five platforms can reveal performance variability linked to the sample through similar trends in performance between all platforms and performance variability linked to an assay through similar trends in performance between all three sample manufacturers for a given variant (see the Methods section and the Data Supplement for further detail). AZ, AstraZeneca Translational Medicine Laboratory; ddPCR, droplet digital polymerase chain reaction; DFCC/Belfer, Belfer Center for Applied Cancer Science at the Dana-Farber Cancer Center; MoCha, Molecular Characterization Laboratory; NGS, next-generation sequencing; NIST, National Institute of Standards and Technology; QCMs, quality control materials.

Each manufacturer provided QCM containing variants at variant allele frequencies (VAFs) of 5%, 2.5%, 1%, 0.1%, and 0% diluted from a common master stock (except Thermo Fisher Scientific *ERBB2* CNV, which was a separate series of diluted reagents). All QCMs were shipped to the NCI Molecular Characterization Laboratory (MoCha) at Frederick National Laboratory for Cancer Research for nucleic acid extraction (assessed by using a TapeStation HSD1000 [Agilent] and Qubit [Thermo Fisher Scientific]), aliquoting, and blinding of variant level identity. All specimens were then sent to each participating laboratory and assayed as described in the Data Supplement (Fig 1).^12^^-^^19^

## RESULTS

**FIG 2.**
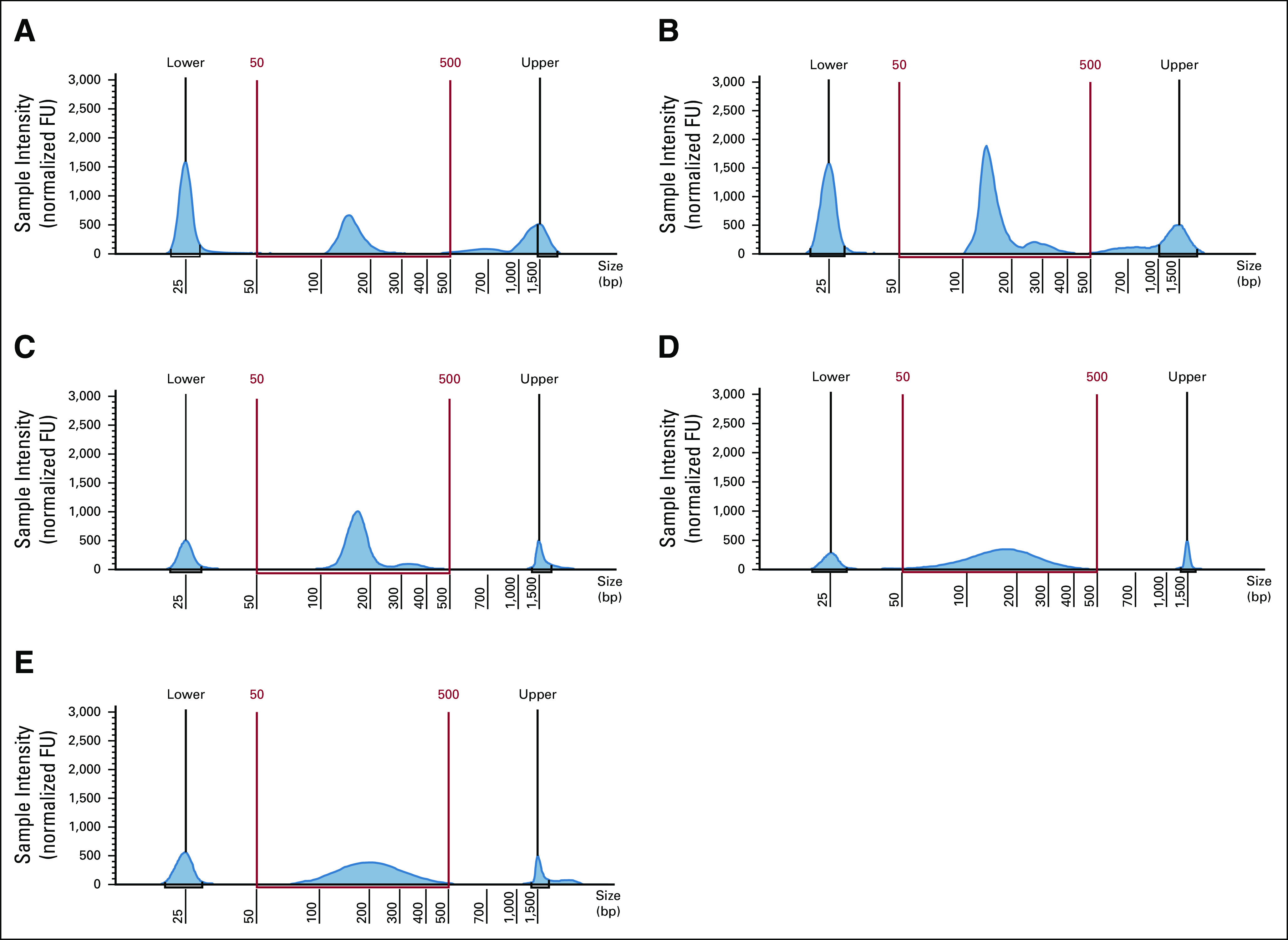
Fragment analysis of the manufactured QCM. TapeStation traces for human clinical and QCM assayed in this study: (A) healthy donor sample (fragment size range 100-300 bp), (B) clinical sample from patient with NSCLC (primary fragment size range 100-250 bp), (C) LGC SeraCare QCM (primary fragment size 75-250 bp), (D) Horizon Discovery QCM (fragment size range 50-500 bp), and (E) Thermo Fisher Scientific QCM (fragment size range 75-500 bp). bp, base pair; FU, fluorescent units; NSCLC, non–small-cell lung cancer; QCM, quality control material.

Size profiles (Fig 2) of QCMs were within the expected cell-free DNA (cfDNA) size range, approximately 150-200 bp, on the basis of TapeStation DNA size measurements. Horizon and Thermo Fisher Scientific QCM had broader fragment distributions than observed for the LGC SeraCare material and healthy donor and lung cancer cfDNA (shown for reference). Distribution patterns from QCM were further validated by sequencing of whole genome libraries at AstraZeneca Translational Medicine Laboratory (AZ; Data Supplement). Each manufacturer used different methods to generate and fragment the QCM, which may contribute to differences in sizing profiles. DNA quantity measurements from TapeStation and Qubit methods were very similar (Data Supplement).

**FIG 3.**
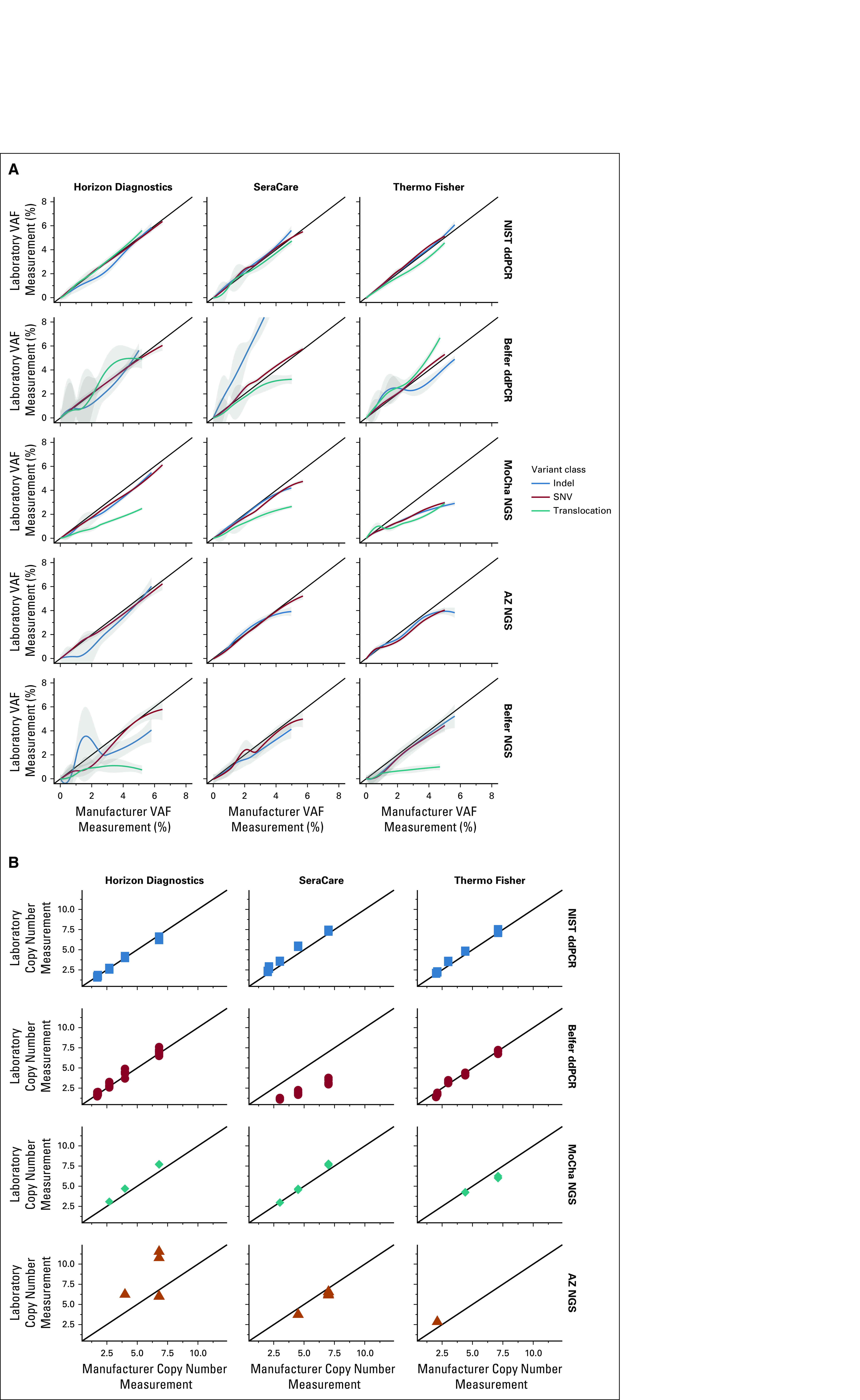
(A) Measured versus manufacturer-specified VAFs. The smoothed average measured VAF (%) versus manufacturer provided VAF (%) aggregated by variant type (ie, SNVs [red], indels [blue], and translocation or fusions [green]) for each of the five participating sites (panel rows) for each of the three QCMs (panel columns). Laboratory measurements for each variant class are summarized as a loess smoothing curve. Corresponding 95% CIs for the average laboratory measurement at any given VAF provided by the manufacture are shown using the gray shaded areas. (B) Measured versus manufacturer-specified *ERBB2* CNV copy numbers. Each replicate is plotted with assay measurements on the *y*-axis and manufacturer values on the *x*-axis. AZ, AstraZeneca Translational Medicine Laboratory; CNV, copy number variant; ddPCR, droplet digital polymerase chain reaction; indel, insertion or deletion; MoCha, Molecular Characterization Laboratory; NGS, next-generation sequencing; NIST, National Institute of Standards and Technology; QCMs, quality control materials; SNV, single nucleotide variant; VAF, variant allele frequency.

For each of the five VAFs, each laboratory performed quadruplicate replicates and reported final data to the statistical team for data assessment. These results were used to assess sensitivity and precision of the variants. Figure 3A depicts the average measured VAF for each variant class versus the expected VAF values provided by the manufacturers. Individual measurements for each specific variant and assay are provided in the Data Supplement.

The average SNV VAFs from all the assays were close to the manufacturers’ reported values. The exception was lower reported VAFs (ie, undercalled) with Thermo Fisher Scientific QCM for all variants tested using the MoCha NGS assay (Data Supplement).

Examination of the QCM indels demonstrates that all measured VAFs compared well with the manufacturers’ reported VAFs, with some exceptions (Fig 3A). The Horizon QCM contained two of the four indels (the *BRCA1* or *BRCA2* indels were not included). The results from Belfer Center for Applied Cancer Science’s *EGFR* exon 19 deletion ddPCR assay for the LGC SeraCare material were 3-fold higher compared with the manufacturer’s claims. This particular QCM actually contained two additional *EGFR* exon 19 deletions in the mixture (COSV51767961 pL747_P753delinsS and COSV51774879 pS752_I759del), and Belfer’s *EGFR* exon 19 deletion detects all exon 19 deletions without determining the exact deletion.^9^ The NGS assays detected the additional *EGFR* variants, but only reported the variant of interest as part of the results. The MoCha NGS assay undercalled the Thermo Fisher Scientific QCM indels. The amplicon NGS assay trend did not appear to be linear across the indel VAF dilutions in the Horizon QCM. This is probably due to the large range of reported VAFs at 1% across the assay replicates (Data Supplement).

There were two translocation targets in the QCM. Each contained *CD74-ROS1* (COSF1200) as well as *EML4-ALK* variant 3 (COSF474; Horizon and Thermo Fisher Scientific) and *EML4-ALK* variant 1 (COSF463; LGC SeraCare). A downward trend with decreasing VAF dilutions was reported for all assays for all QCMs (Fig 3A and Data Supplement). There were a large range of replicate VAF calls from the Belfer ddPCR (Data Supplement and Fig 3A). Notably, both the MoCha and amplicon NGS assays undercalled VAF for both translocations from all manufacturer QCMs, whereas the NIST ddPCR reported VAFs much closer to the VAFs provided by the manufacturers.

Each manufacturer included one CNV for *ERBB2* in the QCM pool. The QCM manufacturers were asked to formulate the equivalent of approximately seven copies of *ERBB2* in the 5% VAF dilution, 4.5 copies in the 2.5% VAF dilution, three copies in the 1% VAF dilution, and 2.1 copies in the 0.1% VAF dilution. The data are shown in Figure 3B and the Data Supplement. Although there was a general trend with higher copies called for 2.1 versus 2 observed using the two ddPCR assays, none of these assays performed accurately compared with the manufacturer’s claims at these values. Only one replicate of the 2.1 copy level was reported by the AZ NGS assay, but all other replicates from all manufactured materials were not distinguished as above two copies by either NGS assay. The Belfer ddPCR and NIST ddPCR reported near the expected copy numbers for three copies and higher, with the exception of the LGC SeraCare reagents, where the target was undercalled by a factor of 2 (Fig 3B). The MoCha NGS reported amplifications at three copies and higher, with the exception of the 3-copy Thermo Fisher Scientific QCM, which failed to generate adequate sequencing libraries in all attempts with both the MoCha and AZ NGS assays. This is likely due to an extraction failure resulting in the low DNA recovery for this particular reagent (Data Supplement). The MoCha NGS did track well with the expected manufacturer’s copy number claims at three copies and above (with the above noted exception). The AZ NGS did not reproducibly detect any QCM below seven copies, but reported a higher copy number with the Horizon QCM compared with the manufacturer’s claims.

**FIG 4.**
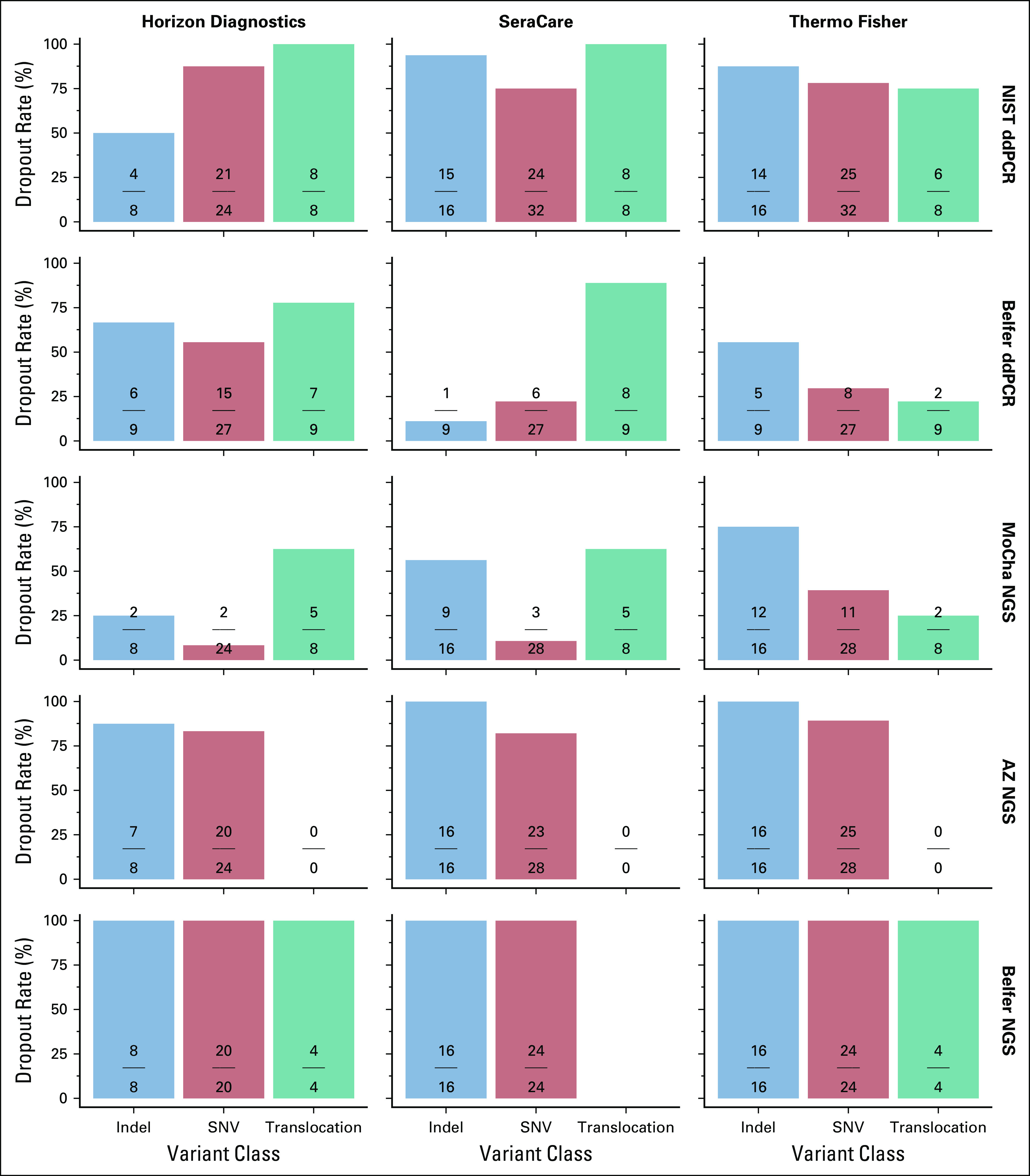
0.1% detection rate for both ddPCR and the three NGS assays. The numerator represents the number of undetected variants, and the denominator represents the total number of tested replicates of all variants within each variant type (indel, SNV, and translocation). Each assay applied the assay-specific reporting limit on the basis of determined limit of detection. No translocations were measured by the AZ NGS test. AZ, AstraZeneca Translational Medicine Laboratory; ddPCR, droplet digital polymerase chain reaction; indel, insertion or deletion; MoCha, Molecular Characterization Laboratory; NGS, next-generation sequencing; NIST, National Institute of Standards and Technology; SNV, single nucleotide variant.

A goal of this effort was to determine if the QCMs could serve as materials to aid in testing assay limit of detection (LOD). Figure 4 depicts the number of nondetected replicates derived from all replicates and all targets within each tested variant class at 0.1% VAF. For example, the data show that no assay detected 100% of each variant type at 0.1%.

**FIG 5.**
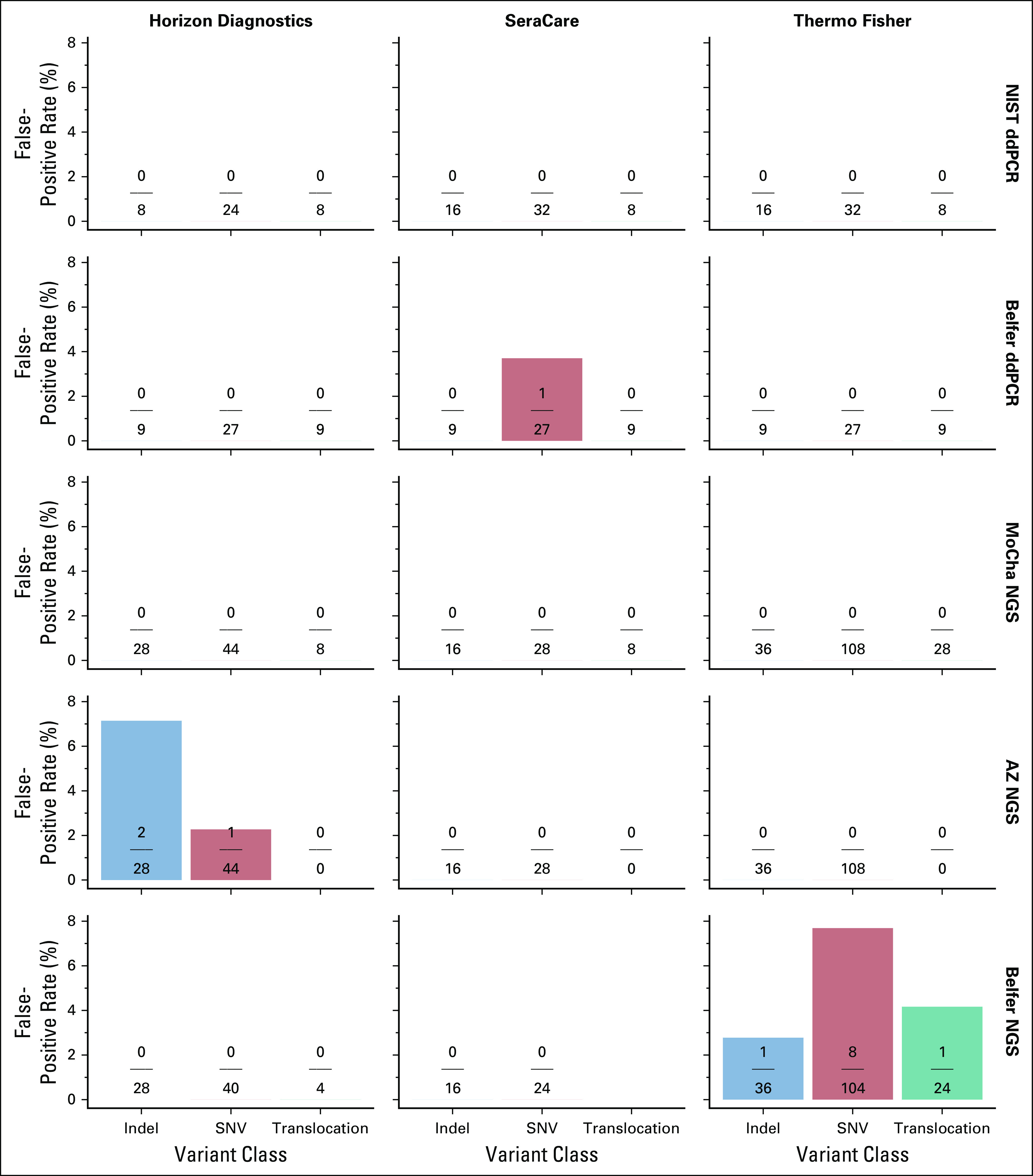
False detection rate-0% VAF specimens. The numerator represents the number of detected variants within each variant class, and the denominator the total number of all tested replicates of each variant within a variant type (indel, SNV, and translocation). Each assay applied the assay-specific reporting limit on the basis of determined limit of detection. AZ, AstraZeneca Translational Medicine Laboratory; ddPCR, droplet digital polymerase chain reaction; indel, insertion or deletion; MoCha, Molecular Characterization Laboratory; NGS, next-generation sequencing; NIST, National Institute of Standards and Technology; SNV, single nucleotide variant; VAF, variant allele frequency.

Data from the 0% VAF specimens indicate the rate of false-positive calls on the basis of the specific and expected variants only, as determined by each assay’s predetermined LOD (Fig 5). Both the NIST ddPCR and MoCha NGS assays have no false-positive calls in the specific variant loci in the three QCMs. The Belfer ddPCR detected an unexpected SNV with the LGC SeraCare QCM. The Belfer and AZ NGS assays had low levels of false-positive calls. The AZ NGS assay reported unexpected positives above the LOD with Horizon reagents in both SNVs (1 of 44, 2.3%) and indels (2 of 28, 7.1%), and the Belfer NGS assay reported unexpected positives with the Thermo Fisher Scientific and LGC SeraCare QCM in SNVs (8 of 104, 7.7%), indels (1 of 36, 2.8%), and translocations (1 of 24, 4.2%).

## DISCUSSION

ctDNA testing requires robust assays that yield accurate and reproducible results. The FNIH ctDNA QCM project was established to test the use of three different QCMs as aids in establishing performance of ctDNA assays. The QCMs containing 14 variant targets at five concentrations were tested in five assays in four different laboratories. Each manufacturer applied different manufacturing methods for target DNA generation and fragmentation. This effort was not intended to test assay analytical performance, but the ability of the QCM to serve as reagents for analytical testing. The data presented demonstrate that the QCMs are capable of providing acceptable reagents for assay performance testing. The results generated from all three QCMs were generally close to expectations compared with the manufacturers’ claims, although several notable unexpected results were observed. The reported results are informative for both the assays and the QCM manufacturers.

The results demonstrate that the two ddPCR assays generally reported VAF values close to the expected results for all variants across the three QCMs. All three commercial manufacturers used ddPCR to qualify and test their reagents. Thus, it is not surprising that the ddPCR assays reported values close to the manufacturers’ claims. The three NGS assays provided some unexpected findings. The NGS assay results showed that SNVs and indels tracked closely to the expected VAF, whereas the translocations did not. Specifically, the two NGS assays that interrogated the translocations reported lower VAFs than expected (resulting in undercalling VAFs) for all the translocations in all QCM mixtures, compared with ddPCR reported VAF values. This may in part be the result of informatic challenges associated with accurate alignment and mapping of hybrid fusion fragments to the human genome. These challenges are overcome by a targeted translocation approach used in ddPCR.

In addition, the MoCha NGS assay reported lower than expected VAFs with the Thermo Fisher Scientific QCM for all variants and variant classes compared with the other two manufactured QCMs. This trend was not observed by either ddPCR or the other two NGS assays. It is likely that fragments bearing variants were carried through the MoCha NGS library preparation step and into the sequencing reaction with less efficiency than their wild-type equivalents. It should be noted that the results still indicate an appropriate downward trend in allele values and replicate precision for the Thermo Fisher QCM was similar to the other QCM.

Detection of a gene amplification in plasma above the large background of wild-type cfDNA from normal tissue can be challenging, especially in cases of low ctDNA fraction or low-level amplification in the tumor. QCMs that are well-characterized with known amounts of an amplified target spiked into a nonamplified normal 2-copy genome may assist assay developers in testing their assays’ ability to detect and report CNVs. The ability to call *ERBB2* copy number amplifications varied widely between assays. The 2.1 copy pool was below the amplification reporting threshold for both NGS platforms and was not accurately discriminated from a copy number of two by both ddPCR assays. ddPCR generally reported all amplifications in the pools. Interestingly, the Belfer ddPCR was the only assay that under-reported CNVs only for the LGC SeraCare *ERBB2* QCM. We can surmise that this result may be attributed to this specific QCM and this specific ddPCR assay, and potential differences in the copy number normalization scheme used by the manufacturer and Belfer ddPCR assays. The AZ NGS assay did not reproducibly call copy numbers < 7 and demonstrated a large range of replicate assay precision in the 7-copy Horizon QCM. The NIST ddPCR and the MoCha NGS reported close to expected values at or above three copies.

Replicate precision data reported from each assay and across all the variant types provide meaningful insights. All technologies and QCM pairings reported acceptable precision, resulting in downward linear trends with decreasing variant target dilutions. An exception was the amplification-based NGS assay, which exhibited the highest variance across most of the variant targets and across all three QCMs.

Dropouts occurred in all assays across all variant types at the 0.1% VAF level (Fig 4). This is an important observation and suggests that the materials from all three manufacturers could serve as materials for formal testing of assay LODs with associated confidence claims for specific variants. An advantage of using a variety of different variants found in these QCMs would help economize the effort to establish statistical claims for an assay. If lower VAF dilutions are required for LOD testing, it is challenging to reproducibly manufacture stochastic amounts of variant targets. A VAF of 0.1% has about 12 variant molecules in a 40 ng input. The DNA input for the NIST ddPCR was 9 ng (approximately 2,700 genomic copies), and the Belfer ddPCR input was 16 ng of DNA. It is reasonable that assays would experience dropouts at the 0.1% VAF (< 3 positives on average expected in 9 ng and five positives in 16 ng of genomic DNA). The NGS assays that input 50 ng of DNA would be expected to have approximately 15 variant targets. This is arguably one of our most important findings. Laboratories are consistently driving LODs lower in an effort to identify potential patient targets. It is imperative that each laboratory tests its LOD with sufficient replicates and dilutions using different targets to make confident claims for LOD. These QCMs provide a sufficient quantity of manufactured variants to assist in assessing LOD. However, LOD claims would need to state that they are relative to the QCM used, as evidenced in the under-reporting of VAFs for the Thermo Fisher QCM with the MoCha NGS assay.

Through the extensive QCM testing performed in phase I, we believe that the data here support the first element of the intended use of the QCM from three commercial manufacturers to aid in the establishment of the performance characteristics of a laboratory’s ctDNA assay. It is imperative that all laboratories become knowledgeable about their own assay technology and the interplay with the QCM to fully understand the strengths and weaknesses of their own reported results.

The next steps of the FNIH ctDNA Quality Control Project, phase II, will focus on functional characterization of these QCMs compared with clinical samples. The study design will focus on determining assay performance with clinical specimens compared with the QCMs to determine if they perform equivalently. If the QCMs demonstrate similar performance, the QCM will be a valuable asset in determining assay analytical performance for variant class sensitivity, LOD, and precision claims by providing contrived specimens containing many variants and in sufficient quantity. Phase III of this effort will involve sharing the QCM with a broader group of commercial and academic clinical laboratories to gather input on the value of the materials to meet the stated intended use as assay controls and a surrogate specimen for assay performance testing.

In conclusion, the overarching goal of this effort is to add rigor to the data that will shape the clinical validity and utility of ctDNA assays for support of cancer patient management. Other notable efforts with similar goals include the Blood Profiling Atlas in Cancer,^10^ which aims to standardize ctDNA data collection and assay validation methods, and NCI’s effort to provide best practices for ctDNA preanalytics.^11^ The roadmap being established in the FNIH QCM project is focused on QCM for ctDNA assays that detect somatic alterations, but we believe that this effort may serve to guide others in the continuing development of useful controls for tumor mutational burden, microsatellite instability, and other biomarkers derived from a liquid biopsy.
